# Spark-Engineered Microelectrode Arrays from Disposable Screen-Printed Macroelectrodes

**DOI:** 10.1021/acsmeasuresciau.6c00191

**Published:** 2026-09-11

**Authors:** Thanawath Tuntiwongmetee, Stéphane Jeanneret, Andrea Nonis, Thomas Cherubini, Yupu Zhang, Apon Numnuam, Suntisak Khumngern, Eric Bakker

**Affiliations:** † Department of Inorganic and Analytical Chemistry, 27212 University of Geneva, Quai Ernest-Ansermet 30, CH-1211 Geneva, Switzerland; ‡ Division of Physical Science, Faculty of Science, 426964 Prince of Songkla University, Hat Yai, Songkhla 90110, Thailand; § Center of Excellence for Trace Analysis and Biosensor, Prince of Songkla University, Hat Yai, Songkhla 90110, Thailand

**Keywords:** Spark engineering, Microelectrode array, Radial diffusion, Steady-state current, Screen-printed electrode, Electrochemical sensor

## Abstract

A computer numerical control (CNC)-guided spark-assisted localized microelectrode patterning is used for the first time for the programmable fabrication of electrodes exhibiting microelectrode behavior with high spatial precision. A graphite wire serves as a spark probe to locally modify a disposable screen-printed carbon electrode (SPCE) overcoated with an insulating polydopamine (PDA) layer formed via electropolymerization. Localized spark discharge can be precisely positioned with three-dimensional (*X*, *Y*, and *Z* axis) CNC control and executed at a rate of ∼1 s per point, giving reproducible patterning of microscale features. While the PDA film effectively suppresses electron transfer, the spark treatment restores electrochemical activity only at predefined microscale positions. The sparked electrodes exhibit increased redox peak currents in cyclic voltammetry and reduced charge-transfer resistance in electrochemical impedance spectroscopy. Chronoamperometric measurements confirm a transition from Cottrellian behavior (unmodified SPCE) to a stable steady-state current (sparked microelectrode), confirming radial diffusion at the exposed micro-openings to the underlying electrode. Combined with precise spatial control, the fabrication of microelectrode arrays with tunable site density is demonstrated, resulting in amplified Faradaic responses and enhanced analytical sensitivity. This approach offers strong potential for advanced electrochemical sensing applications.

## Graphical abstract

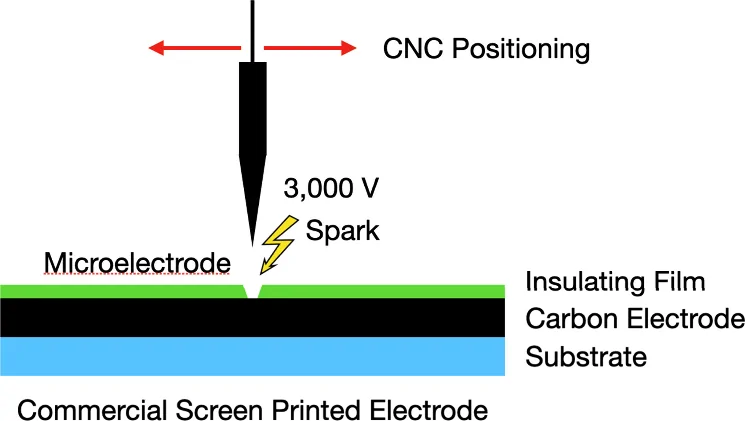

Spark-assisted electrochemical modification has been introduced by the group of Prodromidis as a simple and versatile approach for the modification of electrode surfaces.^1–3^ The electrical discharge between a conductive probe and the electrode substrate can induce localized surface activation and structural transformation under ambient conditions.^1,3^ Such spark discharge forms a rapid, mask-free alternative to conventional microfabrication methods, enabling direct modification of electrochemical interfaces without lithography or vacuum processing.^4,5^ Modern electroanalytical applications greatly benefit from microelectrodes and microelectrode arrays, which however require precision engineering.^6^ Microelectrodes exhibit distinct mass transport characteristics compared to conventional macroelectrodes, most notably the transition from planar to radial diffusion,^7^ which results in steady-state current responses under unstirred conditions, improved signal-to-noise ratios, and enhanced sensitivity.^8^ These features make microelectrodes particularly attractive for fast kinetic studies, trace concentration detection, and operation in complex or confined media, all in the absence of forced convection. However, established fabrication approaches, including photolithography,^9^ thin-film deposition,^10^ and focused ion beam patterning,^11^ are often costly, multistep, and difficult to scale for rapid prototyping or on-demand device fabrication.

We demonstrate here that commercially available disposable screen-printed macroelectrodes can be endowed with microelectrode behavior without a need for advanced microfabrication technologies. A screen-printed carbon electrode (SPCE) was first passivated by forming an electropolymerized polydopamine (PDA) layer, effectively blocking electron transfer. Subsequently, CNC-controlled spark discharge at precise positions gave microscale breakdown of the insulating film, exposing the underlying electrode structure. The process occurs at a rate of ca. 1 s per point and with high spatial reproducibility, allowing for high-throughput patterning of electroactive microdomains that exhibit microelectrode diffusion behavior.

A custom-built CNC-controlled spark instrument with a DC power supply set at 3 kV was used for the fabrication of the microelectrodes through spark-assisted deposition, as shown in Figure S1. A graphite wire (GrW, Pentel, from a local stationery shop in Geneva) was used to generate the spark onto the screen-printed carbon electrode (SPCE) (Metrohm, model DRP-110-U75 with ceramic substrate and a Ø 4 mm carbon working electrode). Insulating PDA films were electropolymerized on the SPCE from 10 mM DA·HCl in 0.10 M phosphate-buffered saline (PBS, pH 7.0) by applying 15 consecutive cycles within a potential window of −0.50 to 0.50 V at a scan rate of 10 mV s^–1^ (Figure 2A).^12^ A decrease in the oxidation peak current was observed at approximately 0.25 V with increasing cycle number, indicating oxidation of DA to dopamine quinone (DAQ)^13^ to form the PDA film. The electrochemical properties of the electrode were investigated using CV with [Fe­(CN)_6_]^3–^ (black line) (Figure 2B), showing a well-defined redox peak with an anodic peak at 0.20 V and a cathodic peak at 0.04 V. These peaks disappeared upon coating with the insulating PDA film (gray line) due to the formation of a poorly conductive layer.^12^

The optical microscope image of the bare SPCE revealed a pristine and relatively uniform carbon ink-printed surface on the electrode substrate^14^ (Figure 1A). After modification with PDA, the electrode surface appeared similar to the bare electrode (Figure 1B). Following the spark approach with 5 sparked points on SPCE using a 0.30 mm diameter GrW, five distinct spark-generated spots were placed on the electrode surface (Figure 1C). The center-to-center distance between adjacent pores was greater than 1.0 mm, ensuring sufficient separation between individual spark-affected regions without overlap. The scheme in Figure 1D illustrates the spark position on the working electrode of the SPCE. The spark locally removed the insulating layer, generating an electroactive microhole with an approximate radius of 42 μm, as observed in the 20× optical microscope image shown in Figure 1E. The SEM image of the spark-induced microhole at 400× magnification (Figure 1F) shows an electrode surface with a roughened surface morphology and layered graphite-sheet-like deposition^15^ within the spark-induced hole region, accompanied by irregular edge features and localized cracking around the spark-affected area. A higher-magnification SEM image at 1000× (Figure S2) provides a clearer view of the deposited graphite sheets and more distinctly delineates the boundary of the spark-induced microhole. The presence of these microcracks is likely associated with the intense localized thermal shock^16^ and rapid energy discharge generated during the spark process.

**Figure 1:**
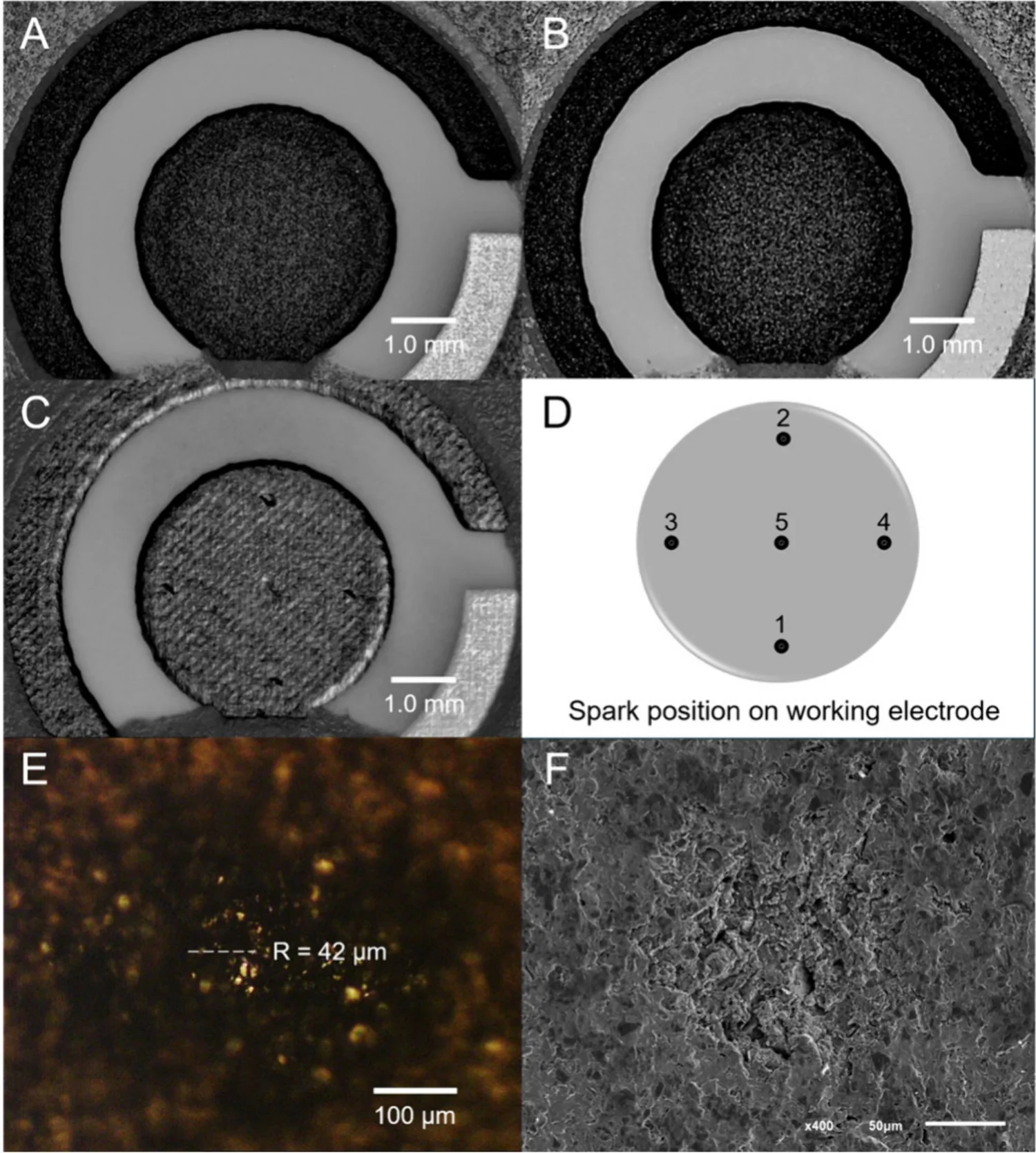
(A–C) Microscope images of (A) SPCE, (B) insulating film/SPCE, and (C) sparked SPCE with intense light at a magnification of 3×. (D) Scheme of sparked microelectrode array with 5 points. (E) Enlarged view of sparked microhole at a magnification of 20×. (F) SEM image of sparked SPCE at a magnification of 400×.

The spark process from GrW is expected to result in deposited graphite-like structures that should adhere strongly onto the microhole. To better visualize the graphite deposition on the electrode surface, a screen-printed gold electrode (SPAuE) was subjected to the same spark treatment without PDA coating to investigate the deposition of graphite from the sparking probe. While the microscope image of the pristine SPAuE shows a clean gold surface (Figure S3A), the spark treatment creates distinct black spots on the gold surface (Figure S3B) that are further visualized by SEM (Figure S3C), confirming graphite deposition.

PDA-insulated SPCE with a single spark gave stable, steady-state currents for the oxidation and reduction processes^8^ in 10 mM [Fe­(CN)_6_]^3–^containing 0.10 M KCl (Figure 2B, blue line) compared to the Cottrell behavior for bare electrode with PDA layer. The spark-generated micrometer-sized openings, surrounded by the insulating PDA layer, act as individual microelectrodes, where radial diffusion occurs, resulting in the observed steady-state current. Impedance analysis shows that the initial charge transfer resistance of 1.6 kΩ for the unmodified electrode increases to 561 kΩ upon PDA deposition, which is reduced to 30 kΩ upon sparking a single microhole. The chronoamperograms exhibited the same trend as cyclic voltammetry (Figure S4; see the Supporting Information for detailed discussion). Locally sparked SPCE activates a single microhole and induces microelectrode behavior.

**Figure 2:**
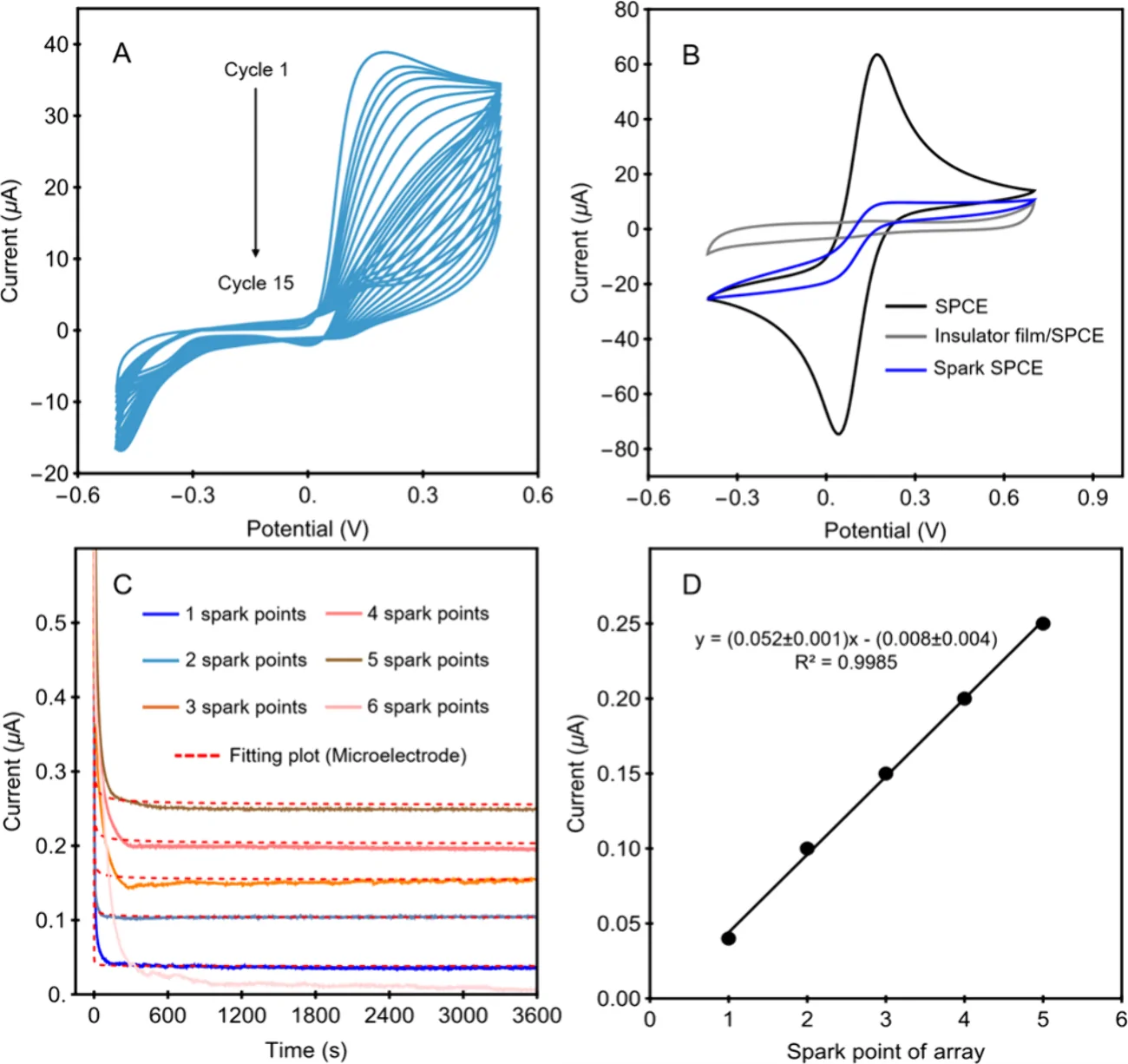
(A) Cyclic voltammograms recorded during the electropolymerization of polydopamine using 10 mM dopamine in 0.10 M phosphate-buffered saline (PBS, pH 7.0) at a scan rate of 10 mV s^–1^ for 15 cycles. (B) Cyclic voltammograms of bare SPCE, insulating film-modified SPCE, and spark-fabricated SPCE recorded in 10 mM [Fe­(CN)_6_]^3–^ containing 0.10 M KCl at a scan rate of 50 mV s^–1^. (C) Chronoamperometric response of the microelectrode array in 10 mM [Fe­(CN)_6_]^3–^ containing 0.10 M KCl (*n* = 3) fitted with the microelectrode-like behavior model. (D) Linear relationship between the current response and the number of spark points of the array.

To evaluate the diffusion regime and the stability of the electrochemical response, the electrochemical behavior of sparked SPCE was investigated chronoamperometrically in a [Fe­(CN)_6_]^3–^ electrolyte under nonstirred conditions over a 1 h period. The absence of *t* ^–1/2^ dependence of the current indicates a clear deviation from planar diffusion to steady-state diffusion behavior after about 60 s (Figure 2C, blue line). The steady-state current can be described by the microelectrode diffusion model with eq 1:^17^

1 $$I=nFADC[{\left(\frac{1}{\pi Dt}\right)}^{1/2}+\frac{1}{r}]$$

where *C* is the bulk concentration of redox species, *r* is the radius of the microelectrode-like holes, and the other parameters have their usual meanings. The spark-induced formation of microscale holes across the insulating film gives rise to radial diffusion that prevents the formation of a growing planar diffusion layer. From fitting to eq 1, the average radius was calculated as 22 μm, which is in reasonable agreement with the observed morphology. The optical radius includes the entire spark-affected and cracked region, whereas the electrochemically estimated radius reflects only the inner conductive area responsible for microelectrode behavior and electrochemical response. Beyond 60 s, the current is found to remain constant for 3600 s, in agreement with microelectrode theory.

The influence of GrW size on the size of the generated microholes was studied with four spark-tip diameters: 0.30 mm (Figure 2C), 0.50 mm (Figure S5A), 0.70 mm (Figure S5B), and 0.90 mm (Figure S5C). Electrodes modified from larger GrW tips exhibited higher current response because of their larger electroactive surface areas. However, only the 0.30 mm GrW sparked electrode demonstrated stable microelectrode-like behavior that closely agreed with the theoretical model over a 1 h period. The larger diameter electrodes induced unstable currents with a pronounced increasing trend as the electrode size increased, suggesting a transition to macroelectrode diffusion behavior.

Microelectrode arrays were fabricated by systematically increasing the number of spark-generated active points (see the images in Figure 1C and the chronoamperometric responses in 10 mM [Fe­(CN)_6_]^3–^ containing 0.10 M KCl in Figure 2C). The currents increase proportionally with the number of activated spark points (Figure 2D), indicating that each additional point contributes independently to the overall Faradaic currents, confirming minimal overlap between adjacent diffusion layers.^18^ EIS gave decreased resistance with increasing numbers of spark points from 30 to 11 kΩ, see Figure S6. We note that the array of six points no longer followed this trend (Figure 2C and EIS in Figure S6), suggesting that an excessive number of sparks may start deteriorating the insulating layer.

The microelectrode array with five sparked points gave comparable responses in stirred and quiescent solutions (Figure S7) that were again maintained for up to 3600 s, indicating the predominance of radial diffusion over external convection. The same array was evaluated for the detection of [Fe­(CN)_6_]^3–^ concentrations ranging from 0.10 to 15 mM at an applied potential of 0.20 V (unstirred conditions; see Figure 3A). The current increased linearly with concentration, as expected (Figure 3B). Repeatability of the 10 mM [Fe­(CN)_6_]^3–^ solution for a single array gave an attractive relative standard deviation (RSD) of 1.3% for five measurements (Figure 3C). After each measurement, the electrode was thoroughly rinsed with ultrapure water, and the subsequent measurement was performed after a 5 min interval to ensure consistent measurement conditions. On the other hand, six different fabricated electrode arrays exhibited good reproducibility, showing a low RSD of 1.1% (Figure 3D). These findings confirm the potential reliability and fabrication consistency of the spark-generated microelectrode array and its promise for electroanalytical applications.

**Figure 3:**
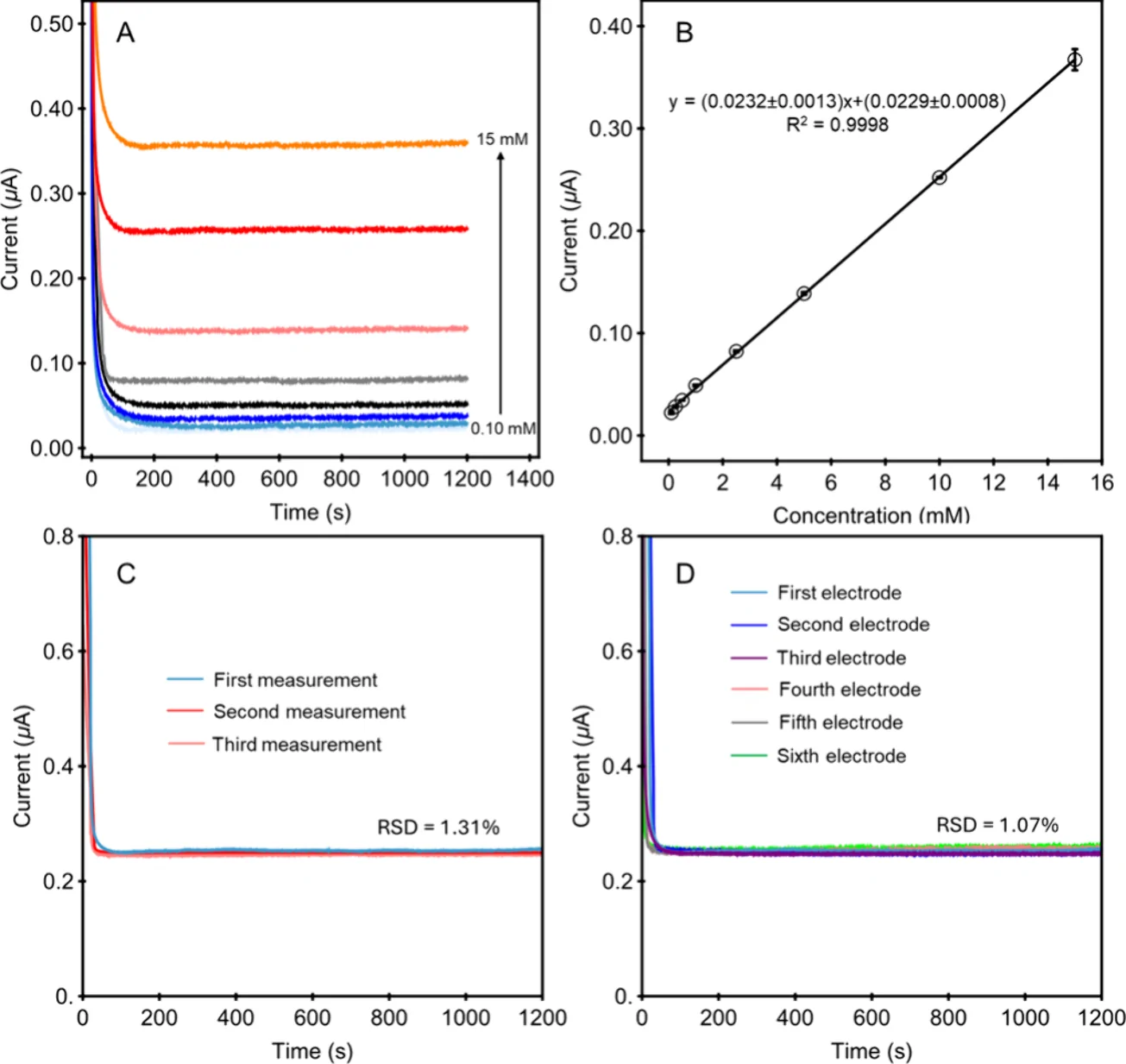
(A) Chronoamperometry of sparked microelectrode array (*n* = 3). (B) Calibration curve for the detection of a series of redox [Fe­(CN)_6_]^3–^ analyte concentrations of 0.10, 0.25, 0.50, 1.0, 2.5, 5.0, 10, and 15 mM. (C) Repeatability of the same electrode in 10 mM [Fe­(CN)_6_]^3–^ containing 0.10 M KCl for three measurements (*n* = 3). (D) Reproducibility of six electrodes (*n* = 6) in 10 mM [Fe­(CN)_6_]^3–^ containing 0.10 M KCl.

The proposed fabrication strategy allows for the precise and controllable generation of multiple spark-induced active sites, providing a simple and rapid method for constructing electrode arrays without the need for complex lithographic processes. The electrochemical characterization confirmed that the fabricated electrodes displayed radial diffusion-control and steady-state responses consistent with microelectrode array behavior and with high repeatability.

## Supplementary Material



## Data Availability

The original data for this work are available athttps://doi.org/10/hccfvk.

## Notes

Microscope and SEM images of bare and sparked electrodes, electrochemical impedance spectroscopy data and associated discussion, chronoamperograms for electrodes sparked with graphite electrodes of larger diameter, and impedance and chronoamperometry for sparked microarray electrodes (PDF)

## References

[1] Hernández-Rodríguez J. F., Trachioti M. G., Hrbac J., Rojas D., Escarpa A., Prodromidis M. I. (2024). Spark-Discharge-Activated 3D-Printed Electrochemical
Sensors. Anal. Chem..

[2] Mertiri M., Hrbac J., Prodromidis M. I., Economou A., Kokkinos C. (2024). Digital fabrication
of 3D printed bismuth sparked sensors for electrochemical sensing. Appl. Mater. Today.

[3] Shetty S. S., Rizalputri L. N., Trachioti M. G., Yuvaraja S., Mani V., Prodromidis M. I., Salama K. N. (2024). Non-destructive integration of spark-discharge
produced gold nanoparticles onto laser-scribed graphene electrodes
for advanced electrochemical sensing applications. Surf. Interfaces.

[4] Takano N., Doeswijk L. M., van den Boogaart M. A.
F., Auerswald J., Knapp H. F., Dubochet O., Hessler T., Brugger J. (2006). Fabrication
of metallic patterns by microstencil lithography on polymer surfaces
suitable as microelectrodes in integrated microfluidic systems. J. Micromech. Microeng..

[5] Li, T. ; Nie, M. ; Morimoto, Y. ; Takeuchi, S. Microelectrodes Fabricated by Vacuum Filling with Low Melting-Point Alloy for Muscle Tissue Stimulation. In 2023 IEEE 36th International Conference on Micro Electro Mechanical Systems (MEMS), January 15–19, 2023; IEEE, 2023; pp 381–383. 10.1109/MEMS49605.2023.10052569.

[6] Tanwar A., Gandhi H. A., Kushwaha D., Bhattacharya J. (2022). A review on
microelectrode array fabrication techniques and their applications. Mater. Today Chem..

[7] Sandison M. E., Anicet N., Glidle A., Cooper J. M. (2002). Optimization of
the Geometry and Porosity of Microelectrode Arrays for Sensor Design. Anal. Chem..

[8] Klymenko O. V., Amatore C., Svir I. (2007). Time-Dependent
Diffusion–Migration
at Cylindrical and Spherical Microelectrodes: Steady- and Quasi-Steady-State
Analytical Solution Can Be Used under Transient Conditions. Anal. Chem..

[9] Liu S.-Y., Liu G., Tian Y.-C., Chen Y.-P., Yu H.-Q., Fang (2007). An Innovative Microelectrode Fabricated
Using Photolithography for Measuring Dissolved Oxygen Distributions
in Aerobic Granules. Environ. Sci. Technol..

[10] Kim N., Hong Y., Cha S., Kang Y. N., Seo H. W., Moon H., Ahn J., Intisar A., Kim M. S., Kim S.-W. (2024). Double-Sided,
Thin-Film Microelectrode Array with Hemispheric
Electrodes for Subretinal Stimulation. Adv.
Mater. Technol..

[11] Doherty J., Ward B., Kellogg E. (1983). Focused Ion Beams in Microelectronic
Fabrication. IEEE Trans. Compon., Hybrids, Manuf.
Technol..

[12] Varol H. S., Herberger T., Kirsch M., Mikolei J., Veith L., Kannan-Sampathkumar V., Brand R. D., Synatschke C. V., Weil T., Andrieu-Brunsen A. (2023). Electropolymerization of Polydopamine
at Electrode-Supported Insulating Mesoporous Films. Chem. Mater..

[13] Wang J.-l., Li B.-c., Li Z.-j., Ren K.-f., Jin L.-j., Zhang S.-m., Chang H., Sun Y.-x., Ji J. (2014). Electropolymerization
of dopamine for surface modification of complex-shaped cardiovascular
stents. Biomaterials.

[14] Romportong S., Numnuam A., Nontipichet N., Tuntiwongmetee T., Thavarungkul P., Kanatharana P., Khumngern S. (2024). Flow injection
amperometric sensor based on reduced graphene oxide modified electrode
for continuous salicylic acid detection. ScienceAsia.

[15] Trachioti M. G., Hemzal D., Hrbac J., Prodromidis M. I. (2020). Generation
of graphite nanomaterials from pencil leads with the aid of a 3D positioning
sparking device: Application to the voltammetric determination of
nitroaromatic explosives. Sens. Actuators, B.

[16] Latella B. A., Liu T. (2006). The initiation and
propagation of thermal shock cracks in graphite. Carbon.

[17] Conceição V. S., Saraiva D. P. M., Denuault G., Bertotti M. (2024). Calibration-Free Analysis
with Chronoamperometry at Microelectrodes. Anal.
Chem..

[18] Reller H., Kirowa-Eisner F., Gileadi E. (1982). Ensembles of microelectrodes:
A digital-
simulation. J. Electroanal. Chem..

