# Supplementary material for: Spark-Engineered Microelectrode Arrays from Disposable Screen-Printed Macroelectrodes
Source: ACS Meas Sci Au. 2026 Sep 11;6(5):1483–8. doi: 10.1021/acsmeasuresciau.6c00191 (PMC13693267; doi:10.1021/acsmeasuresciau.6c00191)
Supplement: Supplementary file 1 [file tg6c00191_si_001.pdf]

Supplementary information

Spark-Engineered Microelectrode Arrays from Disposable Screen Printed Macroelectrodes

Thanawath Tuntiwongmetee[a,b,c], Stéphane Jeanneret[a], Andrea Nonis[a], Thomas Cherubini[a], Yupu Zhang[a], Apon Numnuam[b,c], Suntisak Khumngern[b,c], Eric Bakker[a*]

[a] Department of Inorganic and Analytical Chemistry, University of Geneva, Quai Ernest-Ansermet 30, Geneva CH-1211, Switzerland

[b] Division of Physical Science, Faculty of Science, Prince of Songkla University, Hat Yai, Songkhla, 90110, Thailand

[c] Center of Excellence for Trace Analysis and Biosensor, Prince of Songkla University, Hat Yai, Songkhla 90110, Thailand

Chemicals

Dopamine hydrochloride (DA·HCl), potassium dihydrogen phosphate, and dipotassium hydrogen phosphate were obtained from Sigma-Aldrich (Merck) (St. Louis, MO, USA). Potassium ferricyanide $[Fe(CN)_6]^{3-}$ and potassium ferrocyanide $[Fe(CN)_6]^{4-}$ were obtained from Thermo Fisher Scientific (Geel, Belgium). All chemicals used were of analytical grade. All solutions were prepared in Milli-Q water (18.20 MΩ cm).

Spark-engineered microelectrode approach

The spark instrument system consisted of a high-voltage power supply (Bertan Series 230, USA) integrated with a Computer Numerical Control (CNC; Makerbase, China) positioning stage. The CNC stage was controlled through a custom LabVIEW (National Instruments) interface, enabling precise three-dimensional (X, Y, and Z) positioning. This configuration allowed accurate control of key fabrication parameters, including the initial tip position, spark location, tip-to-substrate distance, and discharge duration. During fabrication, the probe was positioned 2 mm above the substrate and lowered to a sparking distance of 0.5 mm for 1 s. A potential of 3 kV was applied to generate the electrical discharge. Under these optimized conditions, a single spark discharge reproducibly created a microscale hole on the electrode surface, resulting in the fabrication of a spark-engineered microelectrode.

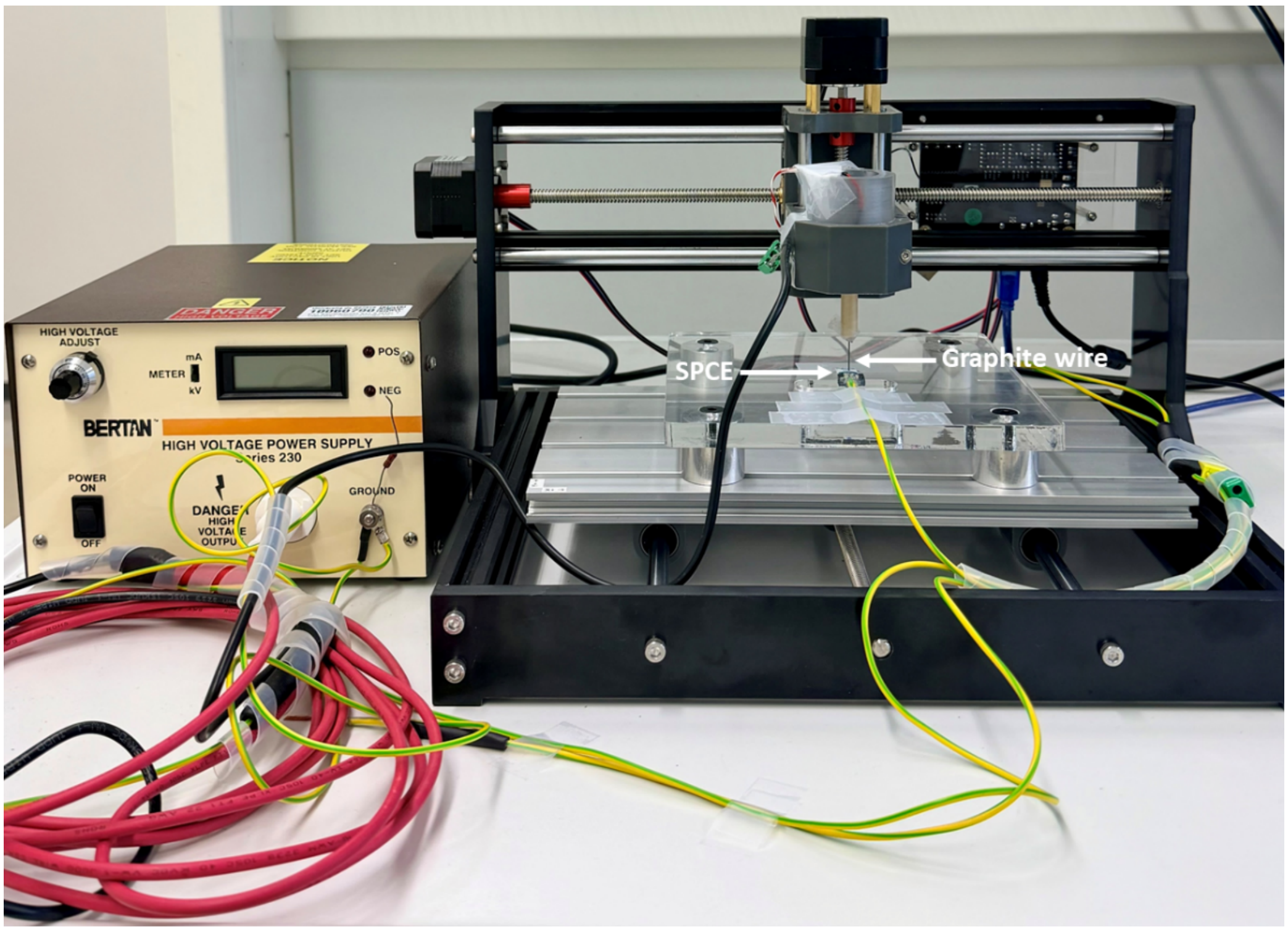

Figure S1. Spark instrument system of CNC coupled with a high-voltage power supply.

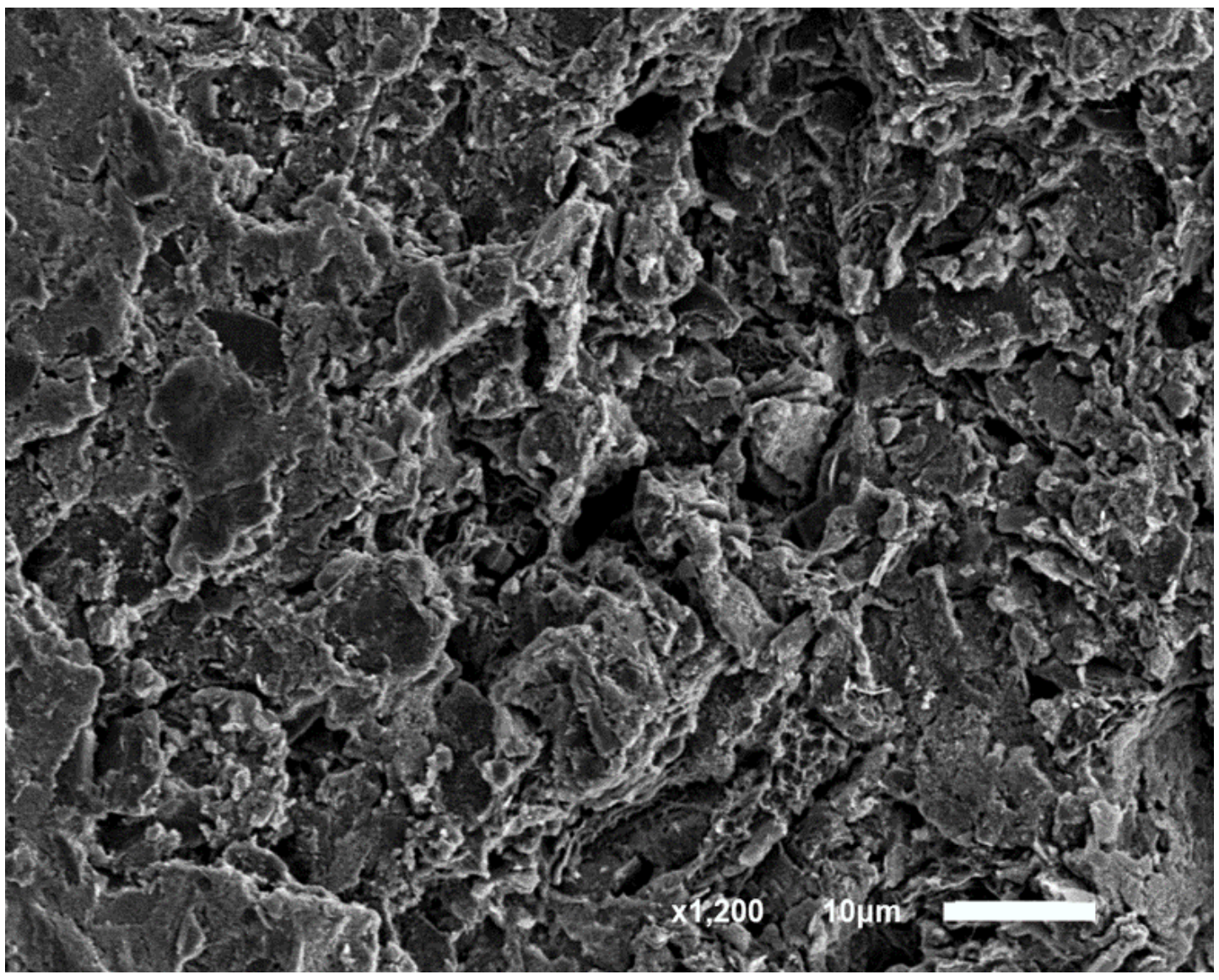

Figure S2. SEM images of sparked SPCE at 1000x magnification.

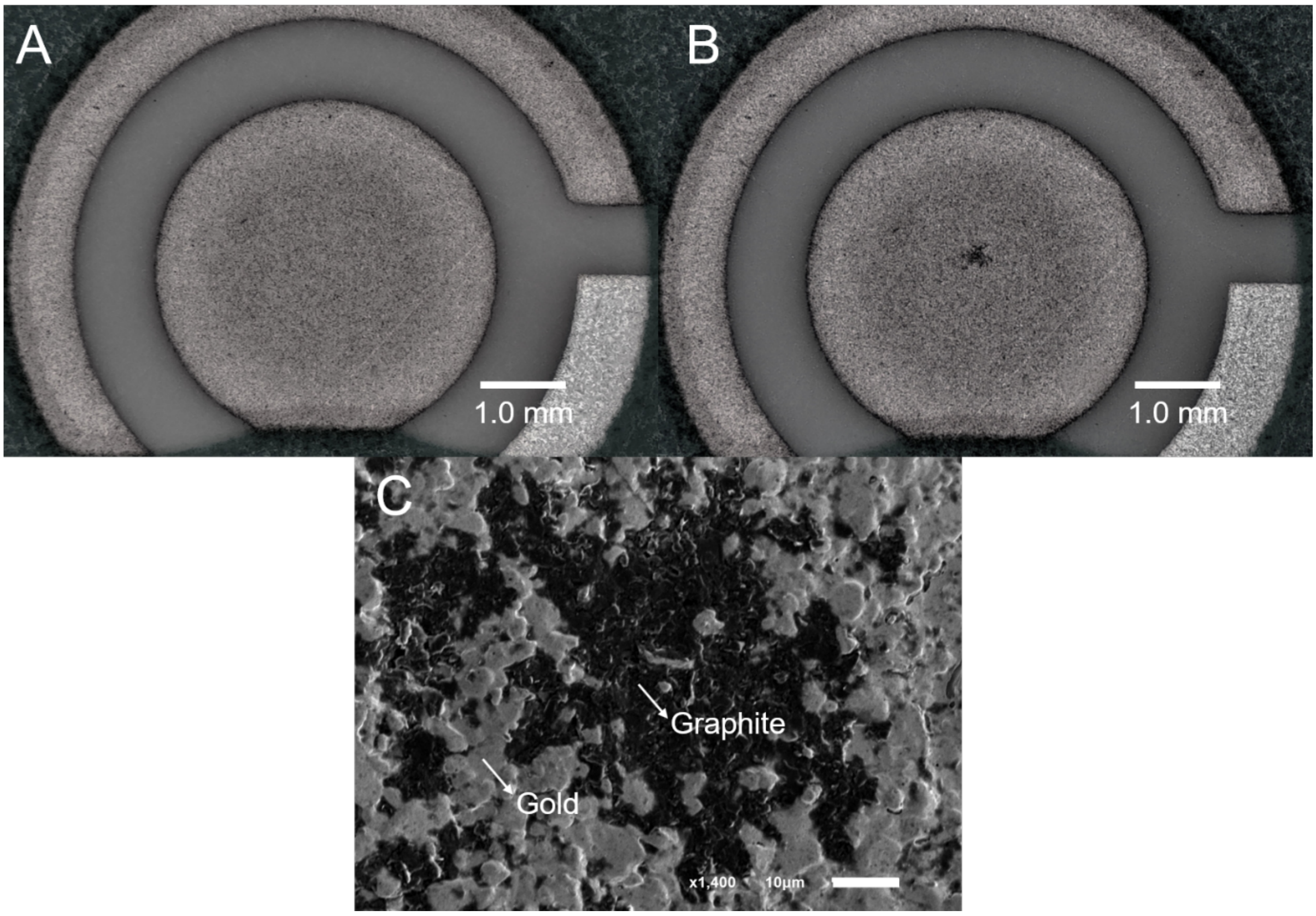

Figure S3. Microscope image of (A) SPAuE and (B) Sparked SPAuE at a magnification of 3×. (C) The SEM image of sparked SPAuE at 1400× magnification.

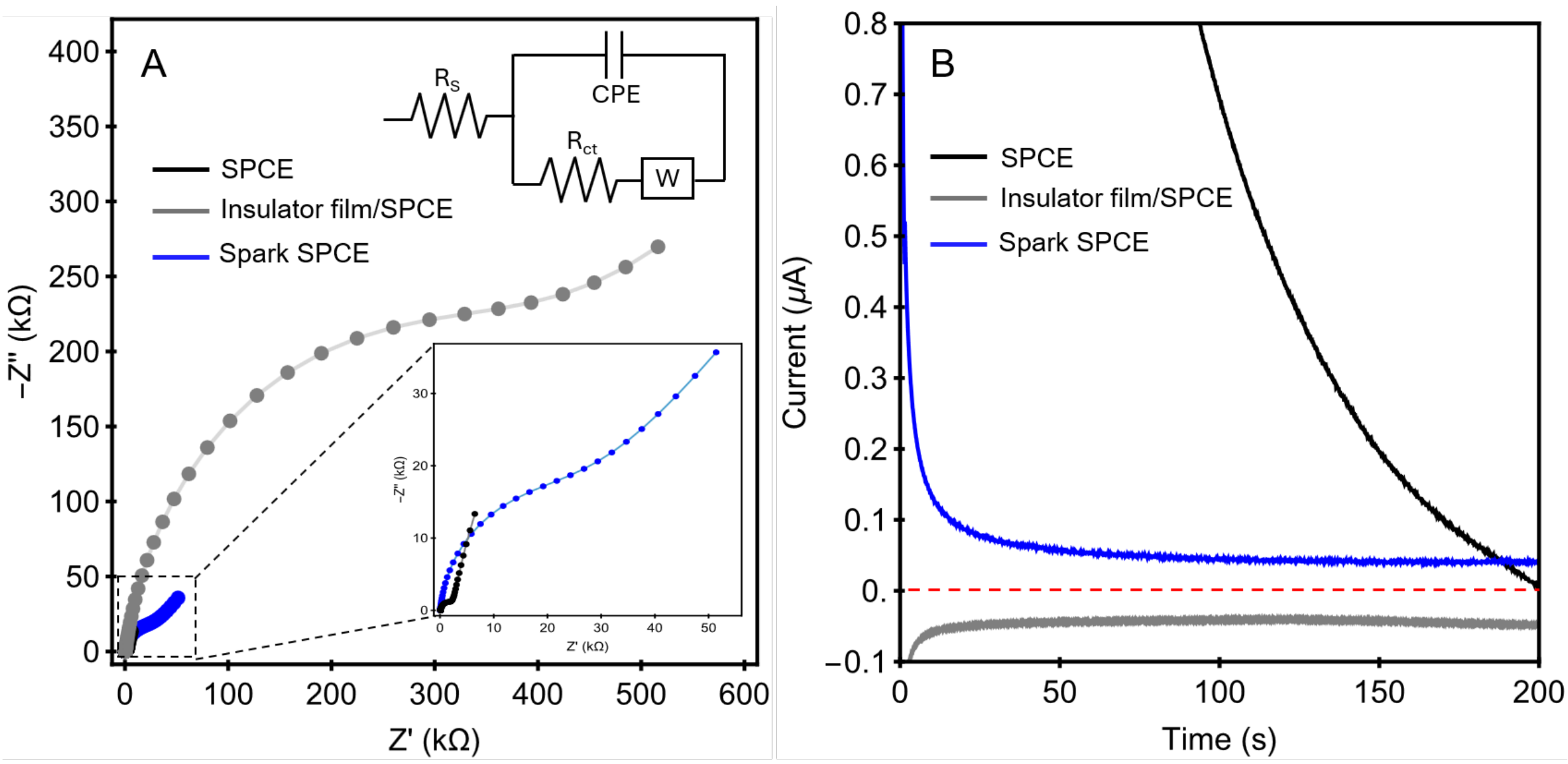

Figure S4. (A) Electrochemical impedance spectra of SPCE, insulating film/SPCE, and sparked SPCE in 10 mM $[Fe(CN)_6]^{3-/4-}$ over a frequency range from 10 kHz to 10 Hz, and (B) Chronoamperograms of SPCE, insulating film/SPCE, and spark SPCE recorded in 10 mM $[Fe(CN)_6]^{3-}$ at a constant potential of 0.20 V for 200 s.

Impedance analysis

For the electrochemical impedance spectroscopy (EIS) analysis, the modified electrodes were further evaluated to confirm their interfacial electrochemical behavior. The EIS was performed at the open-circuit potential (OCP) over a frequency range from 10 kHz to 10 Hz. The obtained Nyquist plots were fitted using a Randles equivalent circuit model, which consists of the solution resistance (R_s), charge transfer resistance (R_{ct}), a constant phase element (CPE) representing the non-ideal double-layer capacitance, and the Warburg impedance (Z_W) associated with ion diffusion and mass transport processes at the electrode interface.[1] The Nyquist plots enabled quantitative comparison of interfacial electron-transfer kinetics among the different electrode surfaces, as reflected by the diameter of the semicircle corresponding to R_{ct}.[2] The bare SPCE (black line) exhibited a semicircular arc at high-to-medium frequencies,[3] followed by a Warburg tail at low frequencies, which is characteristic of a typical diffusion-controlled electrochemical process of the ferric/ferrocyanide ($[Fe(CN)_6]^{3-}$/$[Fe(CN)_6]^{4-}$) redox couple. The R_{ct} value of bare SPCE was 1.6 kΩ, indicating relatively facile electron transfer at the unmodified screen-printed carbon surface due to its inherent conductivity and accessible electroactive sites. In contrast, insulating film/SPCE showed a dramatically enlarged semicircle (gray line), with R_{ct} increasing to 561 kΩ (356 folds of bare SPCE). This substantial increase confirms that the deposited layer severely hindered charge transfer by blocking active sites and increasing the interfacial barrier for electron tunneling.[4] The strong suppression of the faradaic process is consistent with a dominant capacitive behavior and reduced electrochemical accessibility of the electrode surface. Interestingly, spark-modified SPCE exhibited a much smaller semicircle (blue line) compared to the insulating film/SPCE, with a significantly reduced R_{ct} of 30 kΩ (19-fold reduction of insulating film/SPCE). This improvement suggests that although the insulating matrix is present, the spark process induces localized carbonization and incorporation of graphitic-like conductive pathways within the microholes. These conductive domains effectively restored partial electron percolation pathways. From the Randles fitting perspective, the decrease in R_{ct} also implies a reduced interfacial electron-

transfer barrier and improved heterogeneous electron-transfer rate constant.[2,5] Additionally, the more vertical low-frequency line relative to the insulating film/SPCE indicates enhanced ion-diffusion accessibility, likely due to the porous microstructure formed during spark deposition. Overall, these results demonstrate that the spark treatment transforms a highly resistive modified surface into a partially conductive, fast-electron-transfer, and electrochemically active interface, enabling significantly faster electron-transfer kinetics than the purely insulating configuration.

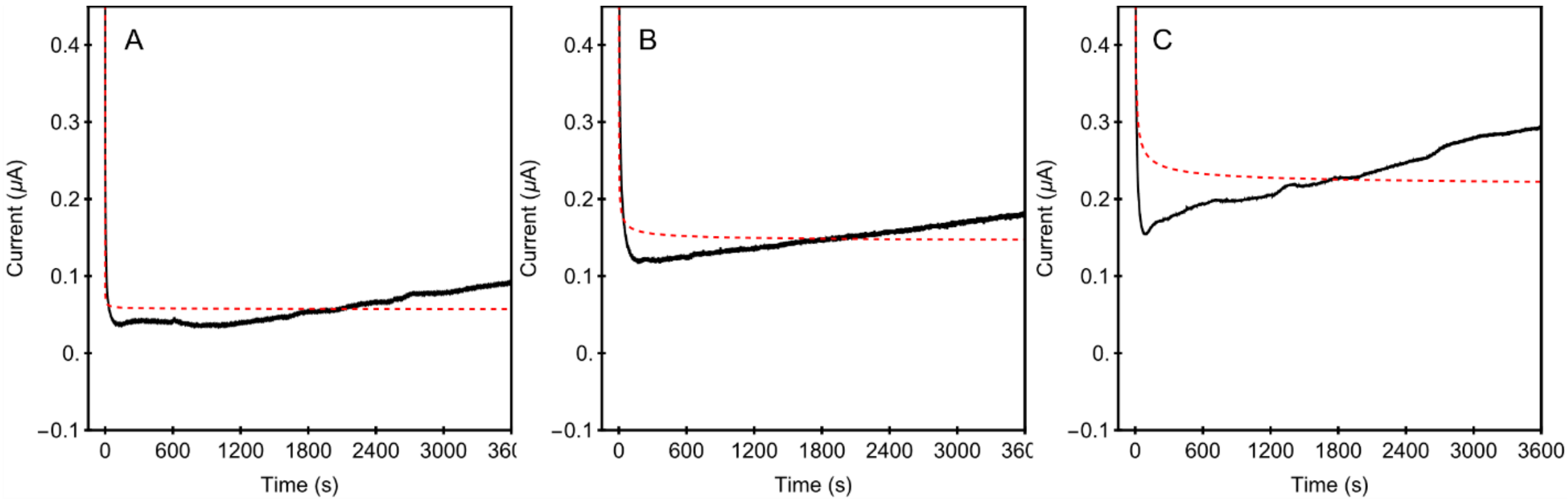

Figure S5. Chronoamperogram of (A) 0.50 mm, (B) 0.70, and (C) 0.90 mm GrW spark electrode with the corresponding fitting of microelectrode-like behavior.

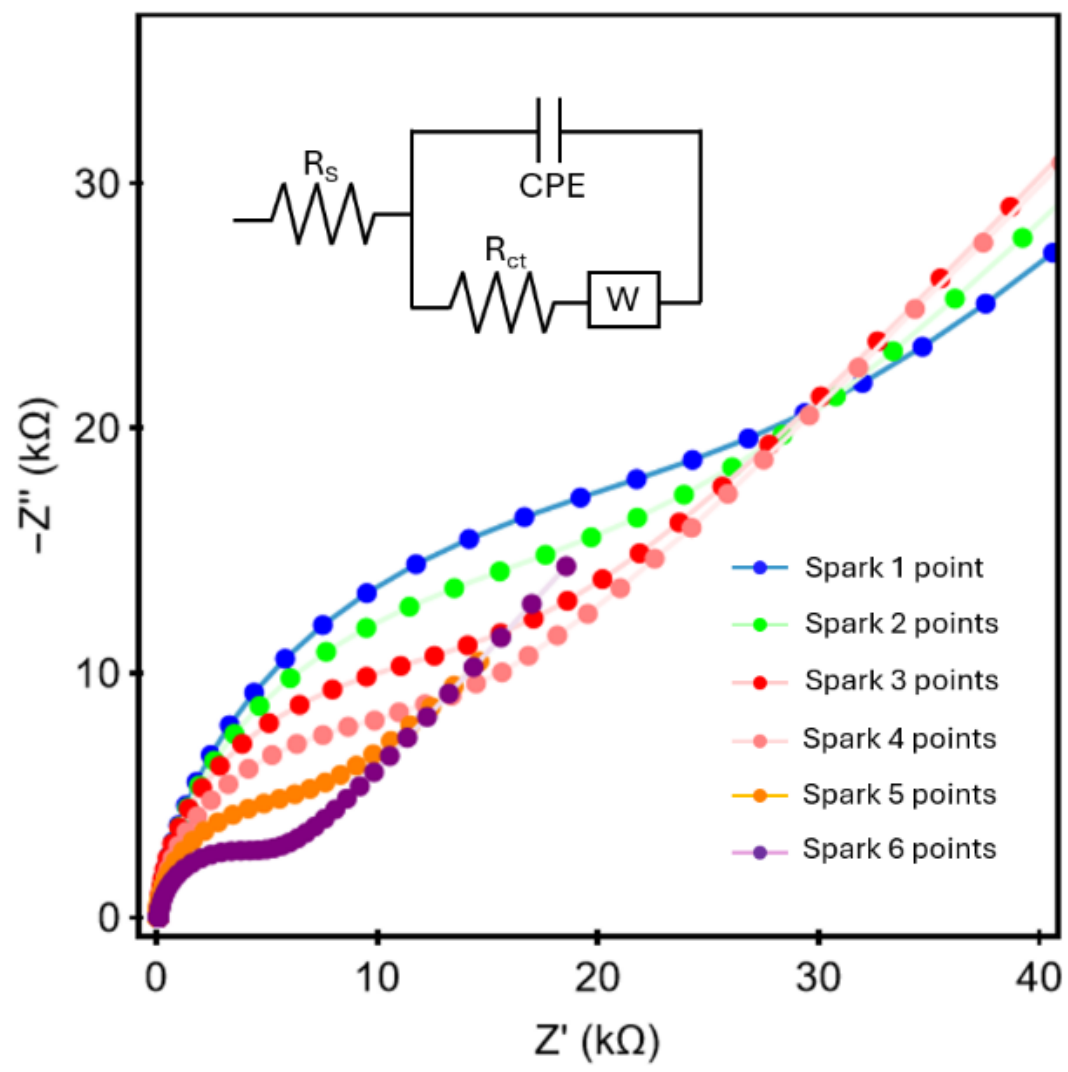

Figure S6. Electrochemical impedance spectra of spark SPCE from 1 to 6 points in 10 mM $[Fe(CN)_6]^{3-/4-}$ over a frequency range from 10 kHz to 10 Hz.

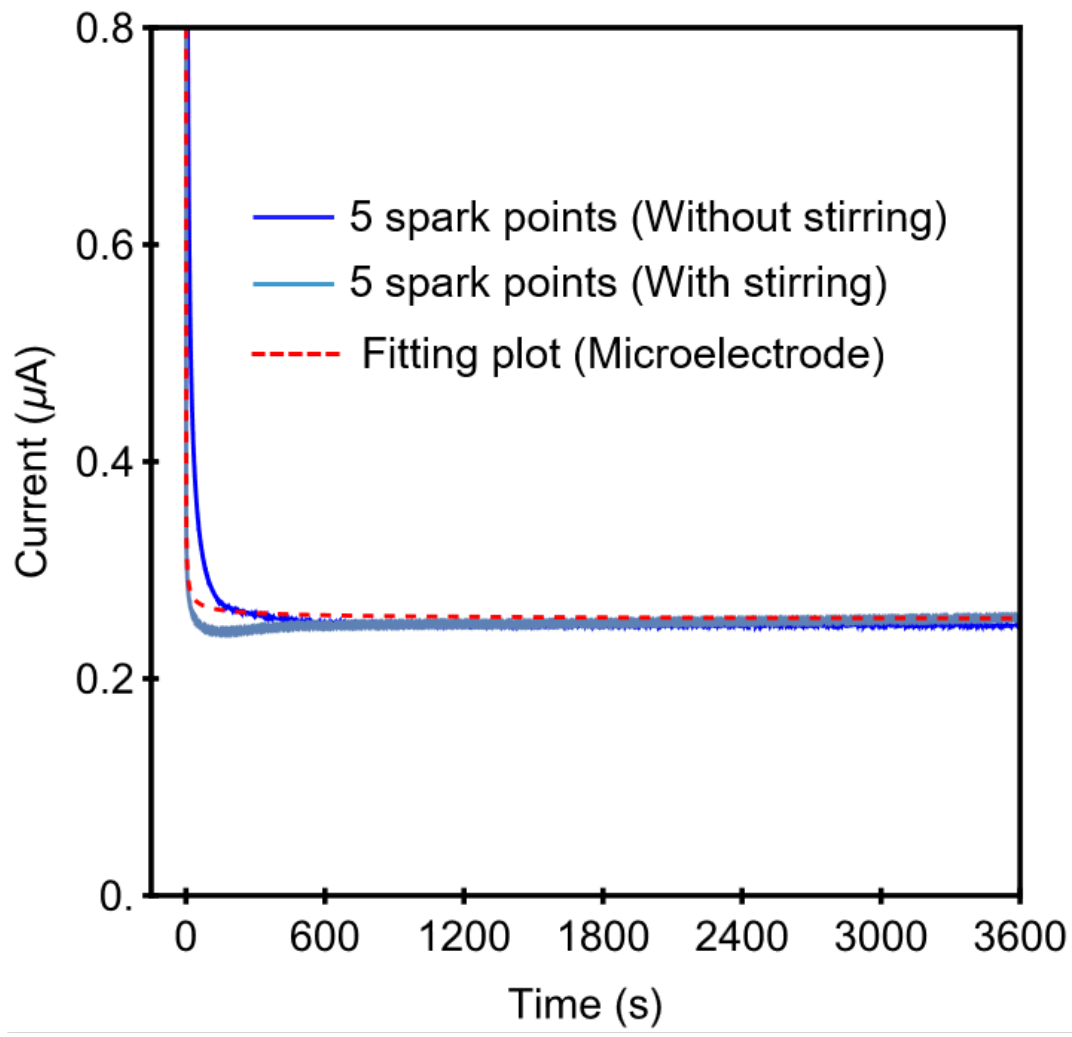

Figure S7. Chronoamperogram of 5 spark points for microelectrode array for the measurement without stirring and with stirring, compared with the fitting of microelectrode-like behavior.

References

(1) Randles, J. E. B. Kinetics of rapid electrode reactions. *Disc. Faraday Soc.* **1947**, *1* (0), 11-19, 10.1039/DF9470100011. DOI: 10.1039/DF9470100011.
(2) Lazanas, A. C.; Prodromidis, M. I. Electrochemical Impedance Spectroscopy─A Tutorial. *ACS Meas. Sci. Au* **2023**, *3* (3), 162-193. DOI: 10.1021/acsmeasuresciau.2c00070.
(3) Verma, H.; Mittal, A.; Kumar, P.; Bhattacharya, B.; Upadhyay, S. Redox-Active La2FeNiO6–rGO Nanocomposites as Electrodes with Tuned Electrochemical Properties for Asymmetric Supercapacitor Applications. *ACS Appl. Energy Mater.* **2026**, *9* (9), 5581-5599. DOI: 10.1021/acsaem.5c04131.
(4) Eloul, S.; Batchelor-McAuley, C.; Compton, R. G. Thin film-modified electrodes: a model for the charge transfer resistance in electrochemical impedance spectroscopy. *J. Solid State Electrochem.* **2014**, *18* (12), 3239-3243. DOI: 10.1007/s10008-014-2662-1.
(5) Hernández-Rodríguez, J. F.; Trachioti, M. G.; Hrbac, J.; Rojas, D.; Escarpa, A.; Prodromidis, M. I. Spark-Discharge-Activated 3D-Printed Electrochemical Sensors. *Anal. Chem.* **2024**, *96* (25), 10127-10133. DOI: 10.1021/acs.analchem.4c01249.
